# Nanoporous Carbon Nanofibers Decorated with Platinum Nanoparticles for Non-Enzymatic Electrochemical Sensing of H_2_O_2_

**DOI:** 10.3390/nano5041891

**Published:** 2015-11-06

**Authors:** Yang Li, Mingfa Zhang, Xiaopeng Zhang, Guocheng Xie, Zhiqiang Su, Gang Wei

**Affiliations:** 1 State Key Laboratory of Chemical Resource Engineering, Beijing University of Chemical Technology, Beijing 100029, China; E-Mails: liyang1113@hotmail.com (Y.L.); zmingfa@hotmail.com (M.Z.); xiaopeng-zhang@hotmail.com (G.X.); guochengxie3@gmail.com (X.Z.); 2 Hybrid Materials Interface Group, Faculty of Production Engineering, University of Bremen, Bremen D-28359, Germany

**Keywords:** electrospining, carbon nanofibers, nanoparticles, biosensor, H_2_O_2_

## Abstract

We describe the preparation of nanoporous carbon nanofibers (CNFs) decorated with platinum nanoparticles (PtNPs) in this work by electrospining polyacrylonitrile (PAN) nanofibers and subsequent carbonization and binding of PtNPs. The fabricated nanoporous CNF-PtNP hybrids were further utilized to modify glass carbon electrodes and used for the non-enzymatic amperometric biosensor for the highly sensitive detection of hydrogen peroxide (H_2_O_2_). The morphologies of the fabricated nanoporous CNF-PtNP hybrids were observed by scanning electron microscopy, transmission electron microscopy, and their structure was further investigated with Brunauer–Emmett–Teller (BET) surface area analysis, X-ray photoelectron spectroscopy, X-ray diffraction, and Raman spectrum. The cyclic voltammetry experiments indicate that CNF-PtNP modified electrodes have high electrocatalytic activity toward H_2_O_2_ and the chronoamperometry measurements illustrate that the fabricated biosensor has a high sensitivity for detecting H_2_O_2_. We anticipate that the strategies utilized in this work will not only guide the further design and fabrication of functional nanofiber-based biomaterials and nanodevices, but also extend the potential applications in energy storage, cytology, and tissue engineering.

## 1. Introduction

Recently, the electrochemical biosensors have attracted more and more attention for their excellent performance in both basic and applied studies [1,2,3]. Among all its applications, the analytical determination of hydrogen peroxide (H_2_O_2_) is an emerging field [4,5,6]. Some techniques, such as spectrophotometry [7], chemiluminescence [8], and electrochemistry [9,10] have been employed previously to detect H_2_O_2_. However, electrochemical detection as a novel nonenzymatic detection method of H_2_O_2_, has always been the research focus due to its simplicity and low cost [11].

In order to ensure the detection performance of nonenzymatic electrochemical sensors, a suitable catalyst is necessary [12,13]. Generally speaking, metal nanoparticles (MNPs) are the major candidates for the fabrication of electrochemical sensors. Many researches have confirmed that Au, Ag, Pt, Cu, and Ni nanoparticles possess very good electrochemical activity toward H_2_O_2_ [14,15,16,17]. Based on this point, many non-enzymatic electrochemical sensors have been fabricated and used for sensing H_2_O_2_ [18,19,20,21,22,23]. For example, in our previous work, we prepared the polyurethane nanofibers (PU-NFs) filled with multi-walled carbon nanotubes (MWCNTs) and silver nanoparticles (PU-MWCNT-AgNPs) by electrospinning. Subsequently, a novel non-enzymatic amperometric biosensor was fabricated by these NFs to detect H_2_O_2_ [18]. In another study, Fratoddi *et al.* prepared a H_2_O_2_ sensor that can work at room temperature by depositing PtNPs onto titania nanofibers (TiO_2_ NFs) obtained by electrospinning [22]. They found that Pt-TiO_2_ hybrid showed combined properties of photoconduction of titania and the photocatalytic activity of the hybrid. Besides, during the sensing tests toward hydrogen, an enhancement of photoconductivity (150%), an increase in response (400%), and an overall improvement of dynamic behavior were observed. Among all the MNPs, platinum nanoparticles (PtNPs) play the most important and irreplaceable roles not only for their excellent electrochemical performances but also for their high stability and activity or oxygen reduction reaction (ORR) [24,25,26]. Previously, we prepared a novel β-phase polyvinylidene difluoride nanofibrous membrane decorated with MWCNTs and PtNPs [25]. Then, the potential applications as electrode material for the fabrication of H_2_O_2_ and glucose biosensors, as well as for oxidation-reduction reaction (ORR) catalysis were further investigated. It was found that the fabricated biosensors are highly stable and sensitive, and can be used for the selective detection of both H_2_O_2_ and glucose. Additionally, the excellent electrocatalysis as ORR catalyst was also displayed.

Electrospinning is a simple but effective technique to synthesize organized functional polymer nanofibers (NFs) with exceptionally long length, uniform diameter, and large surface area [26,27,28,29,30,31]. It has been widely used for the preparation of enzyme-based electrochemical sensors previously [32,33,34,35]. However, only a few studies on the non-enzymatic electrochemical biosensors based on electrospun NFs on electrodes have been reported [36,37]. Carbon nanofibers (CNFs), the newest product of electrospinning, have attracted increasing attention due to their superior chemical, electrical, and mechanical properties [38,39,40]. Among various CNFs, nanoporous CNFs exhibit promising applications in energy conversion and storage, gas adsorption, and biomedical engineering, ascribed to their ultrahigh specific surface area and porosity [41,42].

**Figure 1:**
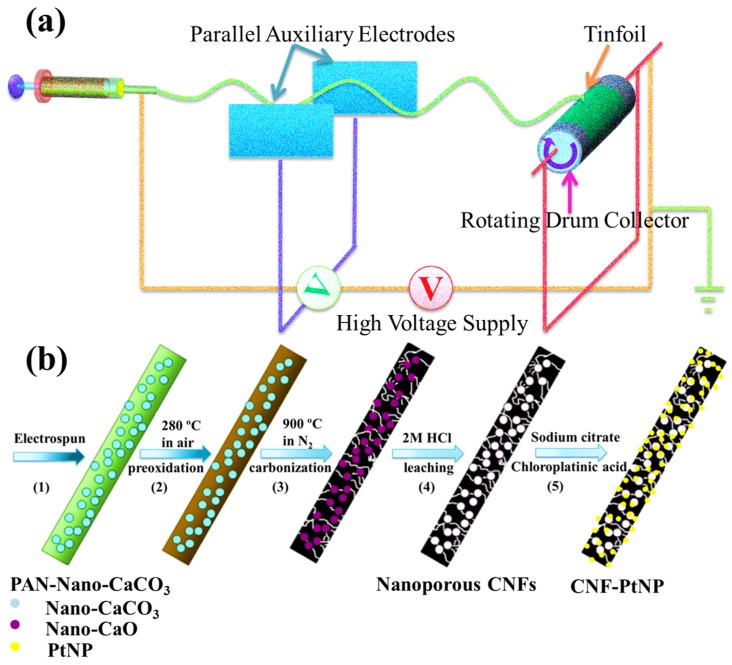
Schematic presentation on: (**a**) The electrospinning apparatus for preparing the polyacrylonitrile (PAN)-CaCO_3_ nanofibers (NFs); and (**b**) Preparation of nanoporous carbon nanofibers (CNFs) decorated with platinum nanoparticles (PtNPs) (CNF-PtNP) hybrid.

In this work, we developed a facile strategy to fabricate a novel nanoporous PAN-based CNF decorated with PtNPs (CNF-PtNP) by electrospinning technique, which can serve as the functional material for the fabrication of non-enzymatic H_2_O_2_ biosensor. Firstly, PAN-CaCO_3_ NFs were electrospun onto the tinfoil, as show in Figure 1a. During the electrospinning process, the porogen (CaCO_3_, nanoscale) can be well dispersed in the created PAN NFs, which can contribute to the formation of NFs with nanoporous structure. After that, PAN-based CNFs were created by the preoxidation and carbonization process, and then nanoporous CNFs were prepared by leaching with HCl (2M). Finally, PtNPs were loaded onto the surface of CNF by the reduction of chloroplatinic acid hydrate (Figure 1b). The fabricated three-dimensional (3D) CNF-PtNP membrane with high surface area ratio to volume is beneficial to the adsorption of electrolytes and the diffusion of reactants. As a result, the special porous structure of the CNFs can result in highly stable, sensitive, and selective detection of H_2_O_2_.

## 2. Results and Discussion

### 2.1. Morphologies of Electrospun Nanoporous CNF-PtNP Hybrids

**Figure 2:**
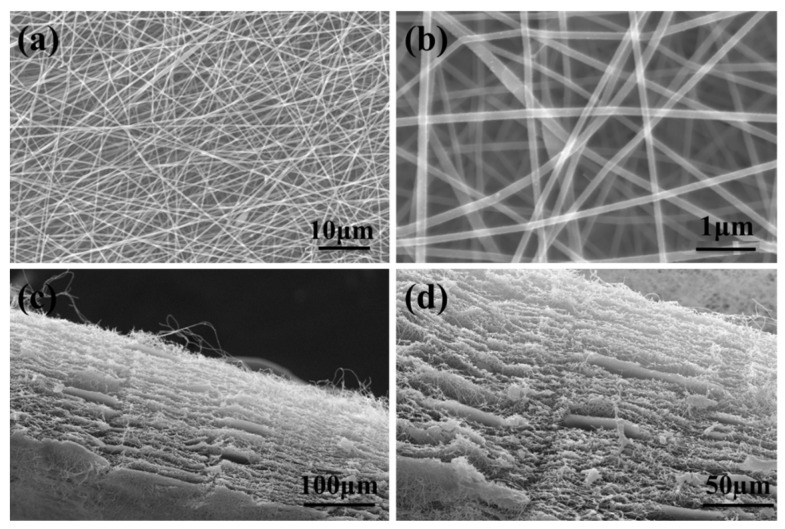
Scanning electron microscopy (SEM) images of electrospun PAN-CaCO_3_ NFs: (**a**,**b**) Different magnification, and (**c**,**d**) Longitudinal section.

The morphologies of the electrospun PAN-CaCO_3_ NFs were observed firstly by scanning electron microscopy (SEM). It can be clearly seen that the created PAN-CaCO_3_ NFs are relatively uniform and uniaxial-oriented with smooth surface, as shown in Figure 2a,b. The width of the created NFs is about 150–300 nm. The dispersion of CaCO_3_ NPs in the electrospun PAN NFs was shown in Figure 2c,d, which is the longitudinal section of the NFs. In this direction, the surface of NFs is rough, on which there are many obvious wrinkles. In addition, some particulate matters of CaCO_3_ NPs can also be observed with a relative uniform distribution (Figure 2d).

**Figure 3:**
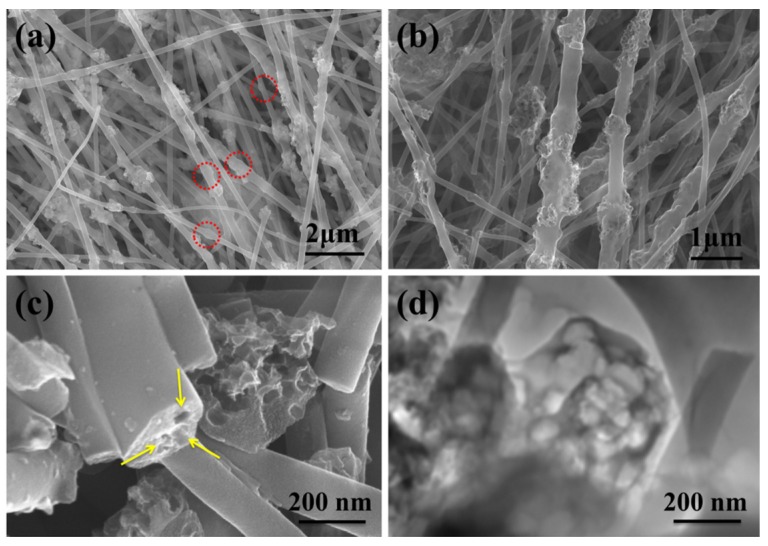
Morphology characterization: (**a**,**b**) SEM images of electrospun PAN-CaCO_3_ hybrid NFs by treating with carbonization and HCl; (**c**) SEM image for the cross-section of porous CNFs; (**d**) TEM image of the porous CNFs.

**Figure 4:**
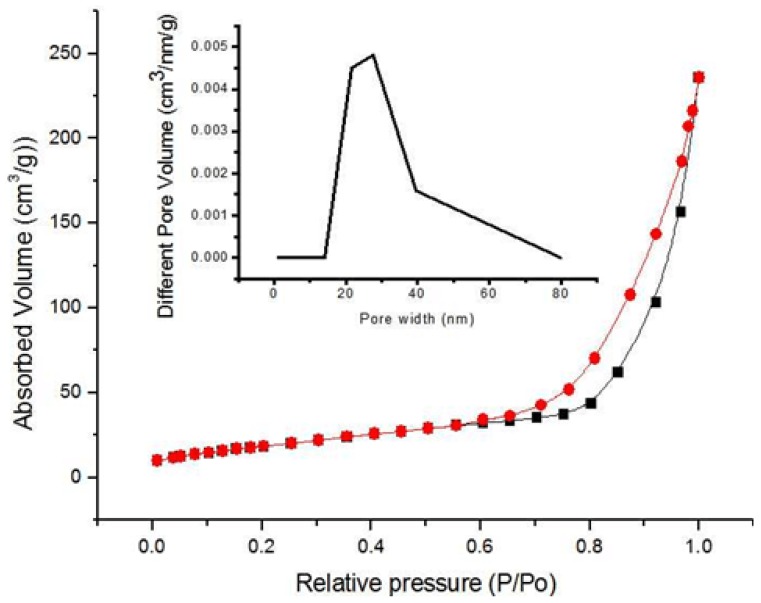
N_2_ adsorption/desorption isotherms of the CNF-PtNP hybrids as well as the pore size distributions.

The morphologies of the electrospun PAN-CaCO_3_ NFs after peroxidation and carbonization are shown in Figure 3a. The integrity of the NFs is almost unchanged, which indicates that the PAN NFs didn’t undergo the oxidative attack severely. After carbonization, CaCO_3_ was decomposed into CaO. While the surface of PAN-CaCO_3_ NFs is much rougher with many CaO nodules distributed throughout the NFs, the distribution of the NFs diameter becomes larger, ranging from about 200 to 400 nm. Due to the high elongation of polymer jet during the electrospinning process, the CaCO_3_ shows a little aggregation and heterogeneous distribution along the fiber axis, as shown in the red circle in Figure 3a. In fact, this kind of CaCO_3_ can be eroded by HCl. Figure 3b shows the typical SEM images of the fabricated nanoporous CNFs, which have been leached by HCl. It can be clearly seen that all the fabricated NFs are highly porous, and the leaching by HCl cannot affect the diameter and length of the electrospun NFs [43]. However, due to the difference of the porogen aggregation, the size of nano-pores embedded in the NFs is not very uniform. The cross-section of the porous CNFs was shown in Figure 3c. As we can see in this figure, the porous structure is very clear. The aggregation of nano-CaCO_3_ in the fiber interior caused by electrospinning might be the source of pores. It should be noted that although the porous structure observed by SEM is not very uniform, the dispersion of nano-CaCO_3_ in the PAN matrix was relatively homogeneous. Figure 3d shows the typical TEM images of the nanoporous CNFs, and it was found that the morphology of the porous structure is very clear. Nitrogen adsorption–desorption isotherm analysis was further performed to evaluate the surface area of the created CNF-PtNP hybrids (Figure 4). The Brunauer-Emmett-Teller (BET) measurement suggests a mecro/mesoporous structure of the created nano-CNFs, as evidenced by the nitrogen adsorption/desorption isotherms of IV type. Based on the BET analysis, the specific surface area of the porous nano-CNFs is calculated to be 131.7 m^2^/g, which is beneficial to the electrochemical performance [44].

### 2.2. Structural Characterization of Nanoporous CNF-PtNP Hybrids

**Figure 5:**
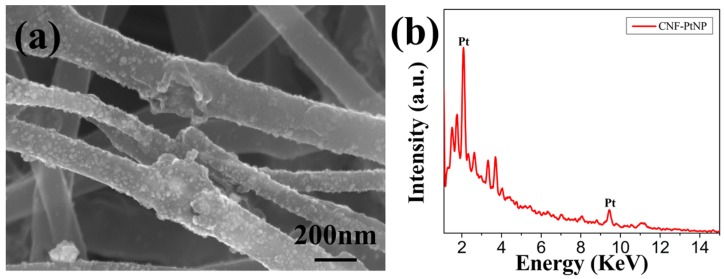
(**a**) Typical SEM image of the fabricated nanoporous CNF-PtNP hybrids; (**b**) Energy-dispersive X-ray (EDX) spectra of the fabricated nanoporous CNF-PtNP hybrids.

Figure 5a shows the SEM image of the created nanoporous CNF-PtNP hybrids. It can be found that the PtNPs were bound onto the surface of CNFs uniformly due to the gentle redox reactions between citric acid and chloroplatinic acid hydrate. The previously generated holes increase the surface area of CNFs dramatically, and therefore cause the adhesion of a large amount of PtNPs onto the CNFs. The typical energy-dispersive X-ray (EDX) spectrum of the fabricated nanoporous CNF-PtNP hybrid is shown in Figure 5b. Two characteristic peaks at about 2.2 and 9.4 KeV on the EDX spectra can be observed clearly, which identifies the successful Pt adsorption on CNFs. By the analysis and calculation of EDX, we can obtain that the adsorption amount of Pt is 8.31%, which is a fairly good adsorption.

**Figure 6:**
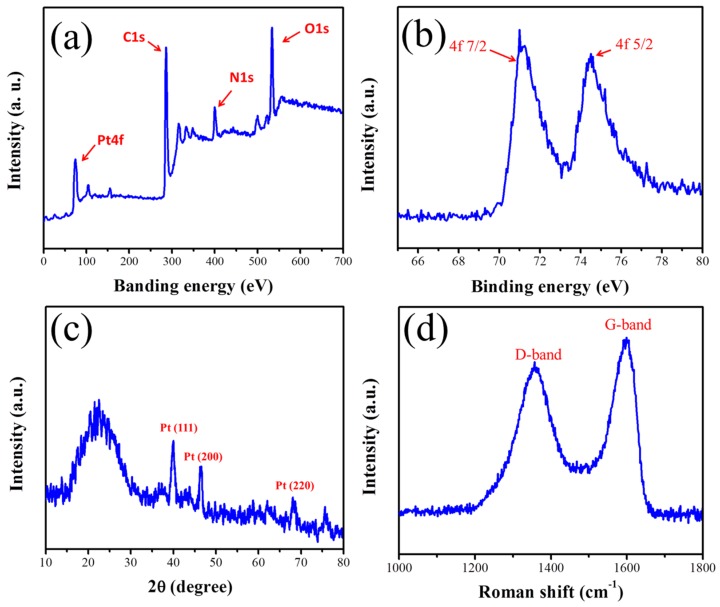
Characterization of electrospun CNF-PtNP hybrids: (**a**,**b**) X-ray photoelectron spectroscopy (XPS) spectra; (**c**) Power X-ray diffraction (XRD) pattern; and (**d**) Raman spectrum.

To identify the existence of PtNPs in the created CNFs, X-ray photoelectron spectroscopy (XPS) was performed firstly. Figure 6a shows the obtained XPS spectrum of the decorated CNFs from 0 to 700 eV. Four different peaks are observed, corresponding to the elemental Pt, C, N and O, respectively. Obviously, the existence of N (9.14%) is the remnant of PAN during the carbonization [45]. To make the peaks of Pt more distinguishable, we carried another XPS analysis with an abscissa from 64 to 80 eV. As a result, two significant characteristic peaks at 71 and 74.3 eV can be seen, which are assigned to the 4f_7/2_ and the 4f_5/2_ planes of the embedded PtNPs [46], respectively. Based on the above XPS results, we suggest that the CNFs are indeed decorated by PtNPs.

Power X-ray diffraction (XRD) was further used to prove the formation of CNF-PtNP nanostructure, and the typical pattern is shown in Figure 6c. Obviously, the peak at 20.2° (C element) of the electrospun NFs is of strongest intensity compared to other peaks. The peaks reveal characteristic reflections of Pt at 39.7°, 46.2°, and 67.5°, which could be assigned to the (111), (200), and (220) planes of the embedded PtNPs, respectively [47] (JCPDS no. 87-0640). Therefore, we can further prove that the electrospun NFs are really the CNF-PtNP hybrids. Figure 6d shows the corresponding Raman spectrum of the fabricated CNF-PtNP hybrids. Two characteristic peaks (D and G bands) at about 1363 and 1594 cm^−1^ [48] can be observed clearly, which identifies the successful carbonization of PAN NFs and the formation of CNFs.

### 2.3. Non-Enzymatic Electrochemical Detection of H_2_O_2_

Considering the good electrocatalytic performance of CNF-PtNP hybrids, as well as their excellent nanoporous structure, the prepared CNF-PtNP hybrids are expected to show good performance for electrochemical sensors. To test this hypothesis, CNF-PtNP hybrids were utilized as the electrode materials to modify the glass carbon electrode (GCE), and the fabricated sensor platform was further utilized for the detection of H_2_O_2_ in this work. To discern the importance of PtNPs on the sensor performances, control experiments with bare GCE, CNF/GCE, and CNF-PtNP/GCE were also carried out.

**Figure 7:**
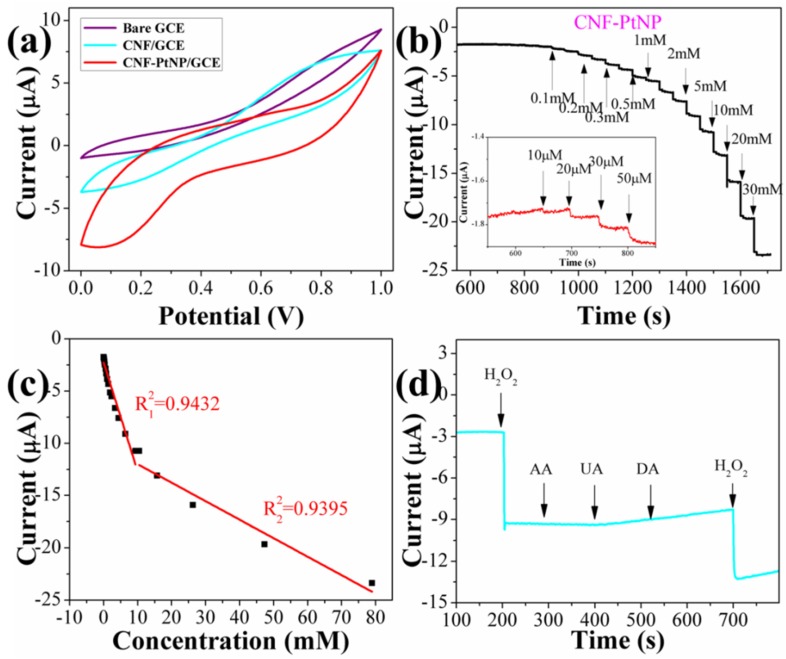
Electrochemical detection of H_2_O_2_: (**a**) Cyclic voltammograms (CVs) of glass carbon electrode (GCE) and GCEs modified with CNFs and CNF-PtNP hybrids; (**b**) I-T response of CNF-PtNP modified GCE; (**c**) Calibrated line; and (**d**) Selectivity of biosensor.

Figure 7a displays the typical cyclic voltammograms (CVs) of bare GCE, CNF/GCE, and CNF-PtNP/GCE in the presence of 5 mM H_2_O_2_. It is clear that both bare GCE and CNF/GCE show no redox processes, while the CNF-PtNP/GCE demonstrates a reduction peak at about 0.4 V with an obvious positive shift for both the onset potential and current peak. Therefore, the present CV results indicate that the CNF-PtNP/GCE has better electrocatalytic activity than other NFs modified GCE toward the reduction of H_2_O_2_, which confirms the importance of PtNPs. To further check the electrocatalytic performance of CNF-PtNP/GCE toward H_2_O_2_, the chronoamperometry of the CNF-PtNP/GCE were also performed at an applied potential of −0.34 V *vs.* SCE. As shown in Figure 7b, a stable response over the long period test and rapid increase in the cathodic current as a result of the reduction of H_2_O_2_ upon adding H_2_O_2_ solutions with different concentrations are observed. The corresponding calibration curve indicates that the sensor shows two linear regions for the response to H_2_O_2_ in the ranges of 10 μM to 9.38 mM and 9.38 to 74.38 mM, as shown in Figure 7c. Based on the first linear range, the line arregression equation of *I* (μA) = −2.2506 − 1.0444 *C* (mM) (*R*^2^ = 0.9432) is determined. In a higher concentration of H_2_O_2_, the second linear section raised up to 90 mM (Figure 6c) with a linear equation of *I* (μA) = −10.3417 − 0.1862 *C* (mM) (*R*^2^ = 0.9395). The limit of detection (*LOD*) of CNF-PtNP/GCE biosensor was determined by using the following Equations [49]:

*LOD* = 3σ⁄*S*

where σ is the standard deviation of the response, and *S* is the slope of the calibration curve.

Compared to previous reports toward the H_2_O_2_ sensor [50], our H_2_O_2_ biosensor based on CNF-PtNP has a similar low *LOD* of about 1.9 μM. Besides, comparing the results shown in Figure 7a, it can be clearly found that the onset potential for H_2_O_2_ reduction in the CNF-PtNP/GCE appears more positive than its counterpart electrode, which reveals the superiority of the electrospun NFs in electrochemical sensing of H_2_O_2_.

Finally, the selectivity and anti-interference capability of the CNF-PtNP/GCE sensor platform were evaluated by amperometry in the presence of ascorbic acid (AA), uric acid (UA), dopamine (DA), which are the common interfering substances for the detection of H_2_O_2_. As shown in Figure 7d, the amperometric responses of the CNF-PtNP/GCE for the successive addition of 1 mM of H_2_O_2_, 0.1 mM of AA, 0.10 mM of UA, 0.10 mM of DA, and 1 mM of H_2_O_2_ were presented. It can be found that the interferences with these species on the detection of H_2_O_2_ are negligible, which means that CNF-PtNP/GCE possesses a high selectivity toward H_2_O_2_.

Based on the above results and discussions, we can deduce that the superiority of this non-enzymatic sensor is ascribed to the advantages of the electrospinning technique. During the electrospinning process, the nanoscale CaCO_3_ can be successfully dispersed and aligned along the NFs, making the fabricated CNFs highly porous, as can be seen from the SEM photographs (Figure 2). In addition, this nanoporous structure can greatly increase the effective adhesion area of PtNPs and further enhance the electrocatalytic activity of electrodes.

## 3. Experimental Section

### 3.1. Reagents and Materials

Calcium carbonate (nano-CaCO_3_) was purchased from Shanxi Xintai Hengxin NanoMaterials Technology Co., Ltd (Shanxi, China). Polyacrylonitrile (PAN) (*Mw* = 150,000, J&K Scientific Ltd., Beijing, China). *N*,*N*-Dimethylformamide (DMF, >99.8% purity) was obtained from Aladdin (Shanghai, China). Sodium citrate tribasic dehydrate (≥99.0% purity), chloroplatinic acid hydrate (≥99.9% purity), and Nafion solution (~5% in a mixture of lower aliphatic alcohols and water) were purchased from Sigma-Aldrich (St. Louis, MO, USA). Disodium hydrogen phosphate (Na_2_HPO_4_), sodium dihydrogen phosphate (NaH_2_PO_4_), ethanol, AA, UA, and DA were purchased from Beijing Chemicals Co., Ltd. (Beijing, China). H_2_O_2_ (analytical grade, 30% aqueous solution) was supplied by Tianjin Dong fang Chemical Plant (Tianjin, China). All chemicals used in this work were analytical reagents and obtained from commercial sources and directly used without additional purification. The water used was purified through a Millipore system (~18.2 MΩ·cm).

### 3.2. Preparation of Nanoporous CNFs

To uniformly generate holes in the PAN matrix, nano-CaCO_3_ was firstly dispersed in DMF using an ultrasonic cleaner operating at 40 kHz for 30 min, respectively. The resulting dispersions were homogeneous and stable. Meanwhile, PAN was dissolved in the mixture of nano-CaCO_3_ and DMF to form a uniform transparent solution by using an ultrasonic cleaner operating at 40 kHz for 1 h. The concentrations of PAN were 3 wt % in the solutions. The dispersions were then loaded into plastic 10 mL syringes with an 18-gauge blunt tip needle and were dispensed at a rate of 0.1–0.3 mL/h during electrospinning. All the samples were electrospun with an applied voltage of 12 kV at a distance of 12 cm from the needle tip to the collector surface of tinfoil. The as-spun PAN NFs were treated by peroxidation at 280 °C in air (at a rate of 2 °C·min^−1^ and keeping this temperature for 2 h). Then a high temperature furnace was employed to stabilize and carbonize the PAN NFs at 900 °C in N_2_ atmosphere (at a rate of 5 °C·min^−1^ to carbonize the NFs, keeping the highest temperature for 2 h). At this time, the nano-CaCO_3_ had been decomposed into the calcium oxide (nano-CaO). Last, HCl was used to leach the NFs. The nano-CaO was dissolved and the porous structure was formed.

### 3.3. Preparation of Nanoporous CNF-PtNP Hybrids

A typical process was employed to load the PtNPs onto the surface of PAN-based nanoporous CNF. In brief, 50 mg CNF and 30 mL chloroplatinic acid hydrate (0.03 mM) were added in a 50 mL flask under a gentle stirring in N_2_ atmosphere at 80 °C, followed by adding 10 mL aqueous sodium citrate (0.1 wt %) react for 1 h to obtain CNF-PtNP. After that, the products were washed with ethanol and distilled water in an ultrasonic bath. Finally, the fabricated nanoporous CNF-PtNP hybrids were dried at room temperature.

### 3.4. Preparation of CNF-PtNP Modified GCE

The procedure for the fabrication of the CNF-PtNP/GCE is as follows: First, 10 mg CNF was added into 10 mL phosphate buffer (pH = 7.6) to obtain CNF suspension under the condition of ultrasonication and stirring. Next, a mixture was created containing 1 mL Nafion (5 wt %) and 2 mL CNF-PtNP suspension, and the mixture was kept stirring for 1 h. Lastly, the CNF-PtNP/GCE was prepared by dropping 10 μL of the mixture onto the surface of a freshly polished glass carbon electrode. The GCE was polished with 1 and 0.3 μm alumina slurry before the modification and then successively washed with ethanol and distilled water in an ultrasonic bath for 10 s respectively. Finally, the fabricated CNF-PtNP/GCEs were dried in air for biosensor application. The dried CNF-PtNP/GCE was kept in storage at 4 °C. Meanwhile, brae GCE and CNF/GCE were prepared as the control experimental. In addition, the mass of CNF and nafion in different electrodes should be kept equal.

### 3.5. Characterization Techniques

SEM morphologies of the electrospun NFs were performed on a JSM-6700F scanning electron microscope (JEOL, Tokyo, Japan) at 20 kV. TEM images were taken by a Tecnai G220 transmission electron microscope (FEI, Beijing, China) with an accelerating voltage of 200 kV, and samples were prepared by directly electrospinning on to the copper grid X-ray diffraction (XRD, Rigaku D/max-2500 VB+/PC, Shanghai, China), X-ray photoelectron spectroscopy (XPS, ThermoVG ESCALAB 250, Tokyo, Japan), and Raman spectroscopy (LabRAM HORIBA JY, Edison, NJ, USA) were used to characterize the structure of samples. The Brunauer–Emmett–Teller (BET, Beijing, China) surface areas of the obtained porous CNFs were analyzed by using nitrogen adsorption in a Micromeritics 3H-2000PS1 nitrogen adsorption apparatus (BET, Beijing, China).

### 3.6. Electrochemical Experiments

All electrochemical experiments were performed on a CHI 660A electrochemical workstation (CH Instruments, Shanghai, China) at room temperature. A conventional three-electrode system was employed with a bare or modified GCE as the working electrode, a Pt wire as the auxiliary electrode, and a KCl saturated calomel electrode (SCE) as the reference electrode. The test solutions were phosphate buffer solutions (PBS, 0.1 M, pH = 7.6), which were prepared with 0.1 M NaH_2_PO_4_ and 0.1 M Na_2_HPO_4_ and deoxygenated with highly pure nitrogen for 20 min before electrochemical experiments. All potentials in this work refer to the SCE. The curves of CVs in this work were obtained after six repetitions of scan numbers under steady-state conditions. Amperometric measurements were carried out under stirred conditions.

## 4. Conclusions

In summary, we demonstrated a facile and efficient electrospinning technique to fabricate novel nanoporous CNFs decorated with PtNPs, and further investigated potential applications, such as H_2_O_2_ biosensors. Electrochemical data indicate that the CNF-PtNP based sensors show good electrocatalytic activity toward H_2_O_2_. The fabricated biosensor shows a wide linear range, low detection limitation, and high selectivity due to the uniform porous structure of CNF, as well as the even coverage of nanoparticles, which are thought to be the main reasons to promote the electrochemical and electrocatalysis properties of the electrospun CNF-PtNP hybrids. We believe that the electrospinning technique can be utilized to prepare nanoporous multifunctional nanomaterials by introducing different functional nanomaterials or building blocks into the polymer NFs as this will be very helpful in fabricating other kinds of sensors with wide applications in actuators, generators, water purification, and energy storage.
